# Progress in Surgery

**Published:** 1895-02-16

**Authors:** 


### Progress in Surgery.

### ORTHOPÆDIC SURGERY.

**The Uses of Localised Application of Dry Air Heated to a High Temperature in Certain Classes of Surgical Affections.**—Mr. A. Willett[1] in a clinical lecture drew attention to this new method of treatment which has been applied to stiffened joints and contracted fasciæ. The method consists in placing the affected limb in a suitable copper cylinder, and exposing it for an hour or more to a temperature of from 200 to 300 deg. Fahr. This process is repeated several times; and it is claimed by the inventor, Mr. Tallerman, that the stiffened structures become so relaxed and pliant after one or two applications that the surgeon can at once painlessly move the part. Mr. Willett has made personal trial of the method in several cases, and could only express a very guarded opinion, mainly because his experience was still very small; yet, small as it was, he had no hesitation in saying it was distinctly encouraging. It seemed to him that the points for consideration were, firstly, the *modus operandi* of the method. He considered this to be the diaphoresis induced by the hot air, and that the closed cylinder acted like a local Turkish bath, and therefore caused an increased flow of blood in the skin and subcutaneous tissues. A remarkable point about the treatment was its anodyne influence, movements that excited pain before can be performed after the limb is placed in the bath without pain; and if adhesions be first broken down under gas and the limb be placed in the bath immediately afterwards the usual pain is diminished or absent. Secondly, the depth from the surface to which the influence of the dry hot-air bath extended. Mr. Willett thought not deeper than the skin and subcutaneous tissue. In firm fibrous articular adhesions, this method gives the surgeon no direct help in obtaining any increase in the range of movement, but whatever movement is present is rendered painless instead of painful.

**Wry-Neck.**—Dr. A. M. Phelps,[2] of New York, had devised an operation, so as to prevent scarring, by beginning the incision at the lobe of the ear. The knife was carried upward, along and behind the ear, following the crease to its superior angle. The incision was then carried directly across into the hair, and then extended obliquely downward, keeping within the hair line, to the side of the neck. This flap was retracted, and the offending sterno-cleido-mastoid cut. Sometimes it was necessary to divide that muscle at its sternal end. The after treatment consists of a brace made of straps attached to a plaster of Paris corset, together with gymnastic exercises.

With care in adjusting sutures and careful antiseptic precautions in performing the operation at the lower part of the neck, the resulting scar is so slight as to be scarcely noticeable, and Phelp's operation is scarcely needed.

**Spinal Caries and its Treatment.**—Mr. A. H. Tubby[3] gives the indications for local treatment as follows: To fix the vertebral column and place it in the best possible conditions for healing; to remove the weight of the upper part of the body from the diseased vertebræ; to prevent unnecessary deformity by supporting the trunk in front; and, if deformity has occurred, to limit its increase. To carry out these indications there are two means at our disposal—recumbency and the use of fixation apparatus. The indications for recumbency are given, and certain data are quoted as to its necessary duration. As to fixation and supporting apparatus, the tests of their efficiency are: (1) They should be so adjusted as to fix firmly the site of disease, and transfer pressure from diseased to healthy spots; (2) they should be comfortable and cleanly; (3) pressure on and chafing of the skin are to be avoided; and (4) they should be such as can readily be applied by the surgeon himself.

**The Application of the Paper Jacket.**—Dr. Marshall Hawkes[4] suggests the use of what is really a papier maché jacket for fixation in spinal lesions. He says it is strong, light, fits very accurately, and a jury-mast can be attached to it without breaking it down.

**"Mallet-Finger."**—R. T. Morris[5] describes this curious deformity. After a blow upon the end of the extended finger, the last phalanx gradually assumes a semi-flexed position, which becomes permanent, the extensor tendon having little or no influence on the phalanx. It is said to be due to rupture of a few fibres of the extensor tendon. It is recommended that the extensor tendon be divided into two bundles and advanced to near the base of the finger-nail, and fixed to the under surface of the skin.

**Congenital Absence of the Entire Tibia.**—Joachimsthal relates the followiug case.[6] A little girl, one and a half years old, came to Professor Wolff's clinic. The right tibia was entirely absent, the foot in a marked position of varus, and the fibula, which was the only bone present in the leg, did not articulate with the femur, but could be felt behind it. The condyles of the femur were very prominent, and the patella could be felt in the intercondyloid notch. The fibula was two and a half centimetres shorter than its fellow, and much thicker. The right buttock was somewhat smaller than the left. The leg was flexed on the thigh. Wolff corrected the varus after tenotomy of the tendo Achillis. By means of a long incision free access was gained to the knee-joint. There were no semilunar cartilages nor crucial ligaments. The head of the fibula was pulled down until it rested between the condyles of the femur, no section being taken from it in order to avoid still further shortening the leg. The capsule was then stitched. As the leg could be completely extended on the thigh after the operation, it was put up in a slightly flexed position in a plaster of Paris bandage, which at the same time retained the foot in good position.

**The Etiology of Deformities Occurring in Knee-joint Disease.**—Phelps[7] deals with the flexion and external rotation which are associated with intra-articular disease. He shows in the first place that the extensors are stronger than the flexors, and that the common notion that the flexors are more powerful is not correct. They only become so when the knee-joint is flexed to relieve pain from tension on the crucial ligaments. If the knee is extended the extensors more than counterbalance the action of the flexors, but immediately flexion begins the mechanical advantage of the flexors increases and that of the extensors decreases, and the greater the flexion the greater the mechanical advantage of the flexors. This can be illustrated by a simple mechanical contrivance. The external rotation is due to the greater power of the biceps over the inner hamstrings.

**Hallux Rigidus.**—Mayo Collier[8] points out that this condition occurs more often in males than females. The affected foot is an abnormally long foot, it is nearly always cold, damp, and more or less blue. The metatarsal bone is flexed on the tarsus and adducted to the mid-line from its fellows; the proximal phalanx is slightly flexed and the head of the metatarsal bone is enlarged; flexion of the joint is generally possible, but extension causes acute pain. Collier gives the results of close and prolonged observation on seven cases. He states that the condition is due to caries on the under aspects of the head of the metatarsal bone at the points of contact with the sesamoid bones. By open incision into the joint and removal of the head of the metatarsal bone excellent results were obtained.

**Club-Foot.**—A most exhaustive account of the anatomy of club-foot has appeared in the "Annals of Surgery," of March, 1894, the author being Dr. Frank Hartley; excellent illustrations are given, and the article merits careful attention.

**Bone Operations for Club-Foot.**—H. A. Wilson[9] gives a careful analysis of 435 bone operations for the correction of club-foot. There were seven deaths: three from septicœmia, three from diarrhœa, and one from carbolic acid poisoning. In one case the foot had to be amputated for gangrene, and two cases are described as failures without any explanation. The age of the patients ranged from three weeks to forty-seven years. Simple excision of the astragalus was done a hundred and fifty-six times, and 68 forms of bone operation were practised on the remaining cases. As to results, 250 were good, in 150 they are not definitely stated, 42 were not benefited, including two amputated for pain and seven deaths. The author expresses his firm opinion that cases of congenital club-foot properly treated from birth would never require any form of tarsectomy.

**Practical Points in the Treatment of Talipes.**—Luke Freer,[10] after discussing the various methods of tenotomy, states emphatically that plaster should never be employed in the after-treatment of talipes. He urges the value of daily manipulations begun from the first, and these should be personally supervised by the surgeon. Tarsectomy should never be done in young children, and only used as a last resource in adults. This paper will well repay perusal.

**Morton's Disease.**—Gibney,[11] in place of excision of the head of the fourth metatarsal bone for this condition, suggests that a boot so made as to raise the arch of the foot well, with a good broad heel and plenty of room in the toes, will be found to give adequate relief. He quotes six successful cases.

**Sweating Feet and Flat Feet.**—Von Lesser[12] claims that sweating feet and flat feet are intimately associated together, the abnormal sweat-production preceding the flat feet. It is quite possible that both conditions may be due to perverted nerve influence.

**Bending of the Neck of the Femur in Adolescence.**—Royal Whitman[13] read a paper, the object of which was to call attention to a deformity at the hip-joint that developed in adolescence under the same conditions as knock-knee, bow-leg, or flat-foot. The trochanter is elevated about Nélaton's line, the leg is rotated outwards, and adduction and inward rotation of the limb are limited. At a later stage there is marked limp from shortening of the leg. He recommends in the early stages extension of the limb, and in the later stages osteotomy below the trochanter.

**Osteoclasis for Rhachitic Deformities.**—At the last meeting of the American Orthopædic Association, Dr. Coolidge,[14] of Chicago, demonstrated the action of the Lorenz osteoclast. With this instrument osteoclasis rivalled osteotomy in its exactness. It is possible to fracture a bone within a small portion of an inch of the desired point. In most cases the fracture was subperiosteal. It could also be used for connecting the deformity of genuvalgum. In either case the skin was never broken.

Mr. R. W. Murray[15] refers to manual osteoclasis in children under five years of age. In 1893 he straightened 311 legs, the total number since 1888 being 778. Not only are curved tibiæ but also knock-knees and outward rotation of the lower limb corrected. He states that the only osteo-clast he ever uses are the hands, and gives details as to the method. His cases appear to have been remarkably successful.

**Operative Treatment of Old Unreduced Dislocations of the Hip.** — Mr. L. Harris[16] advances the following points—(1) That, owing to the danger of fracturing the neck of the femur, laceration of the great vessels of the thigh, shock, and death, the application of great force to reduce old dislocations of the hip should be discontinued in favour of arthrotomy; (2) that subcutaneous operations in old dislocations are without benefit; (3) that osteotomy below the trochanter is of little value; (4) that resection is only to be thought of when reduction by arthrotomy fails. Harris recommends the following procedure: An incision four or five inches long is made vertically between the posterior border of the tensor vaginæ femoris and the gluteus medius. The head and the neck of the femur are denuded, together with a division of all the muscular attachments into the great trochanter and shaft as far down as the lesser trochanter. The muscular attachments are better separated subperiosteally as much as possible, so that when they regain their attachments there will be less derangement. This denudation must be thorough, so that the entire upper end of the femur is perfectly free, otherwise it will be impossible to bring the head of the bone opposite the acetabulum, owing to the contraction of the soft parts. The very close proximity of the great sciatic nerve to the head in posterior dislocations should not be forgotten. After freeing the upper end of the femur, the acetabulum, if filled, should be excavated with a sharp spoon or gouge and mallet. No pains should be spared in the use of the gouge to enlarge the cavity sufficiently to permit the easy entrance of the head. The head is then returned to the acetabulum, the wound closed, extension applied, and the limb immobilised in a slightly abducted position.

**Congenital Displacement of the Hip.**—Kirmisson[17] states that very rarely can the deformity be reduced by manipulation alone. He has treated seven cases by the open method; two were fatal, in two others the results were unsatisfactory, while in three they were very encouraging, as there has been in each after a long interval no tendency to a return of the luxation, and the shortening of the limb has not been more than half an inch. The operation is indicated for patients between the ages of seven and ten years. Articles on the same subject appear in the "Annals of Surgery,"[18] by E. H. Bradford and T. Halsted Myers. Bradford's conclusions are, that the method of treatment by traction, with or without tenotomy, does not effect a cure; correction by means of forcible reduction, without incision, can be applicable in but few cases, and is not reliable; the method of operative reduction offers the best method of cure. Dr. Myers' article is very exhaustive, and he discusses especially the operative aspect of the question, dealing with Hoffa's, Lorenz, and Kirmisson's views. He mentions the dangers of the different methods, and expresses his own views as to the mode of operative reposition. He says that in his opinion the number of perfect cures is very small, the number of cases improved is large; the results in double dislocation are not so favourable as in single, the lordosis is generally corrected, and a slight spinal curvature generally persists, owing to the atrophy of the limbs and pelvis.

[1] Clin. Jour, May 30, 1894. [2] New York Medical Record, Aug. 4, 1894, p. 143. [3] The Hospital, July 14, 1894. [4] New York Medical Record, Aug. 11, 1894. [5] Medical News, Sept. 19, 1893. [6] Annales D'Orthopédie, March, 1894. [7] New York Medical Record, Aug. 4, 1894. [8] Lancet, June 30, 1894, p. 1,614. [9] Amer. Med. and Surg. Bulletin, Feb. 1, 1894. [10] Birmingham Medical Review, July, 1894. [11] The Journal of Nervous and Mental Diseases, Sept., 1894. [12] Amer. Jour. Med. Sci., June, 1894, p. 722. [13] New York Medical Record, June 16, 1894, p. 764. [14] Ibid., Sept. 29, 1894, p. 409. [15] Brit. Med. Jour., Aug. 25, 1894, p. 413. [16] Annals of Surg., Sept., 1894. [17] Revue d'Orthopédie, No. 3, 1894. [18] August, 1894.

